# Supplementary material for: Pathways to housing stability and viral suppression for people living with HIV/AIDS: Findings from the Building a Medical Home for Multiply Diagnosed HIV-positive Homeless Populations initiative
Source: PLoS One. 2020 Oct 1;15(10):e0239190. doi: 10.1371/journal.pone.0239190 (PMC7529314; doi:10.1371/journal.pone.0239190)
Supplement: S1 Table — (DOCX) [file pone.0239190.s001.docx]

**S1 Table. Study sample attrition analysis, HRSA/SPNS** **Building a Medical Home for Multiply-Diagnosed HIV-positive Homeless Populations initiative from 2013-2017**.

| **Characteristic** | **Overall**  **N (%)** | **Not in PATH Analysis**  **N (%)** | **In PATH Analysis**  **N (%)** | **P-Value** |
| --- | --- | --- | --- | --- |
| **Gender**  Male  Female  Transgendered or other | 909  683 (75.1%)  186 (20.5%)  40 (4.4%) | 438  330 (75.3%)  86 (19.6%)  22 (5.0%) | 471  353 (74.9%)  100 (21.2%)  18 (3.8%) | **0.5970** |
| **Race/ethnicity**  Hispanic  Non-Hispanic White  Non-Hispanic Black  Other | 906  174 (19.2%)  253 (27.9%)  411 (45.4%)  68 (7.5%) | 435  75 (17.2%)  130 (29.9%)  199 (45.7%)  31 (7.1%) | 471  99 (21.0%)  123 (26.1%)  212 (45.0%)  37 (7.9%) | **0.3887** |
| **Age**  30 or younger  31-54  55 or older | 909  150 (16.5%)  640 (70.4%)  119 (13.1%) | 438  78 (17.8%)  302 (68.9%)  58 (13.2%) | 471  72 (15.3%)  338 (71.8%)  61 (13.0%) | **0.5644** |
| **Education**  Less than HS  High School  More than HS | 907  285 (31.4%)  302 (33.3%)  320 (35.3%) | 437  134 (30.7%)  151 (34.6%)  152 (34.8%) | 470  151 (32.1%)  151 (32.1%)  168 (35.7%) | **0.7356** |
| **Housing status - baseline**  Homeless  Controlled Environment  Unstably Housed | 909  683 (75.1%)  84 (9.2%)  142 (15.6%) | 438  342 (78.1%)  43 (9.8%)  53 (12.1%) | 471  341 (72.4%)  41 (8.7%)  89 (18.9%) | **0.0184** |
| **Recent incarceration (past 12 months)**  Yes  No | 718  266 (37.0%)  452 (63.0%) | 357  142 (39.8%)  215 (60.2%) | 361  124 (34.3%)  237 (65.7%) | **0.1322** |
| **Physical injury**  Yes  No | 905  398 (44.0%)  507 (56.0%) | 435  193 (44.4%)  242 (55.6%) | 470  205 (43.6%)  265 (56.4%) | **0.8202** |
| **Sexually assaulted**  Yes  No | 906  367 (40.5%)  539 (59.5%) | 438  173 (39.5%)  265 (60.5%) | 468  194 (41.5%)  274 (58.5%) | **0.5491** |
| **Mental health diagnosis prior to enrollment**  Yes  No | 808  641 (79.3%)  167 (20.7%) | 361  280 (77.6%)  81 (22.4%) | 447  361 (80.8%)  86 (19.2%) | **0.2643** |
| **Social support score**  Mean (Std. Dev.) Min, Median, Max | 907 11.2 (5.2) 5.0, 10.0, 25.0 | 436 11.1 (5.2) 5.0, 10.0, 25.0 | 471 11.3 (5.2) 5.0, 10.0, 25.0 | **0.4903** |
| **Change in social support score**  Mean (Std. Dev.) Min, Median, Max | 626 0.6 (6.1) -19.0, 0.0, 20.0 | 156 0.5 (6.7) -17.0, 0.0, 18.0 | 470 0.6 (5.8) -19.0, 0.0, 20.0 | **0.8381** |
| **Self-efficacy score: Getting information** Mean (Std. Dev.) Min, Median, Max | 907 8.7 (2.3) 1.0, 10.0, 10.0 | 437 8.5 (2.4) 1.0, 10.0, 10.0 | 470 8.8 (2.2) 1.0, 10.0, 10.0 | **0.1102** |
| **Self-efficacy score: Obtaining Help**  Mean (Std. Dev.) Min, Median, Max | 908 5.7 (2.4) 1.0, 5.5, 10.0 | 437 5.7 (2.5) 1.0, 5.5, 10.0 | 471 5.7 (2.4) 1.0, 5.5, 10.0 | **0.9047** |
| **Self-efficacy score: Communicating with Physician**  Mean (Std. Dev.) Min, Median, Max | 908 8.7 (2.1) 1.0, 9.7, 10.0 | 437 8.7 (2.1) 1.0, 9.7, 10.0 | 471 8.7 (2.1) 1.0, 9.7, 10.0 | **0.7056** |
| **Change in self-efficacy score: Getting information** Mean (Std. Dev.) Min, Median, Max | 627 0.1 (2.6) -9.0, 0.0, 9.0 | 157 0.1 (2.8) -9.0, 0.0, 9.0 | 470 0.1 (2.5) -9.0, 0.0, 9.0 | **0.7636** |
| **Change in self-efficacy score: Obtaining Help**  Mean (Std. Dev.) Min, Median, Max | 628 0.6 (2.7) -7.5, 0.3, 9.0 | 157 0.4 (2.4) -5.5, 0.0, 7.3 | 471 0.6 (2.7) -7.5, 0.3, 9.0 | **0.2616** |
| **Change in self-efficacy score: Communicating with Physician**  Mean (Std. Dev.) Min, Median, Max | 628 0.3 (2.4) -9.0, 0.0, 9.0 | 157 0.3 (2.0) -6.0, 0.0, 9.0 | 471 0.3 (2.5) -9.0, 0.0, 9.0 | **0.9442** |
| **Health insurance**  Yes  No | 905  575 (63.5%)  330 (36.5%) | 436  278 (63.8%)  158 (36.2%) | 469  297 (63.3%)  172 (36.7%) | **0.8919** |
| **Food insecurity**  Yes  No | 904  530 (58.6%)  374 (41.4%) | 433  256 (59.1%)  177 (40.9%) | 471  274 (58.2%)  197 (41.8%) | **0.7724** |
| **Food needs met**  No  Yes | 906  490 (54.1%)  416 (45.9%) | 436  337 (77.3%)  99 (22.7%) | 470  153 (32.6%)  317 (67.4%) | **<.0001** |
| **Need: Medication assistance**  Yes  No | 908  481 (53.0%)  427 (47.0%) | 437  231 (52.9%)  206 (47.1%) | 471  250 (53.1%)  221 (46.9%) | **0.9475** |
| **Need met: Medication assistance**  No  Yes | 909  539 (59.3%)  370 (40.7%) | 438  339 (77.4%)  99 (22.6%) | 471  200 (42.5%)  271 (57.5%) | **<.0001** |
| **Need: Mental health treatment**  Yes  No | 908  550 (60.6%)  358 (39.4%) | 437  273 (62.5%)  164 (37.5%) | 471  277 (58.8%)  194 (41.2%) | **<.0001** |
| **Need met: Mental health treatment**  No  Yes | 907  607 (66.9%)  300 (33.1%) | 437  361 (82.6%)  76 (17.4%) | 470  246 (52.3%)  224 (47.7%) | **<.0001** |
| **Need: Substance abuse treatment**  Yes  No | 908  394 (43.4%)  514 (56.6%) | 437  212 (48.5%)  225 (51.5%) | 471  182 (38.6%)  289 (61.4%) | **0.0027** |
| **Need met: Substance abuse treatment**  No  Yes | 909  459 (50.5%)  450 (49.5%) | 438  330 (75.3%)  108 (24.7%) | 471  129 (27.4%)  342 (72.6%) | **<.0001** |
| **Number of unmet needs** Mean (Std. Dev.) Min, Median, Max | 909 3.6 (2.3) 0.0, 3.0, 11.0 | 438 3.7 (2.3) 0.0, 4.0, 11.0 | 471 3.4 (2.3) 0.0, 3.0, 11.0 | **0.0332** |
| **Number of barriers to care** Mean (Std. Dev.) Min, Median, Max | 909 3.4 (3.3) 0.0, 3.0, 20.0 | 438 3.6 (3.5) 0.0, 3.0, 20.0 | 471 3.2 (3.1) 0.0, 3.0, 13.0 | **0.0726** |
| **Risk for substance use: Alcohol**  Low  Moderate-High | 909  534 (58.7%)  375 (41.3%) | 438  256 (58.4%)  182 (41.6%) | 471  278 (59.0%)  193 (41.0%) | **0.8601** |
| **Risk for substance use: Cocaine**  Low  Moderate-High | 909  435 (47.9%)  474 (52.1%) | 438  187 (42.7%)  251 (57.3%) | 471  248 (52.7%)  223 (47.3%) | **0.0027** |
| **Risk for substance use: Opioids**  Low  Moderate-High | 909  713 (78.4%)  196 (21.6%) | 438  341 (77.9%)  97 (22.1%) | 471  372 (79.0%)  99 (21.0%) | **0.6797** |
| **Risk for substance use: Amphetamines**  Low  Moderate-High | 909  584 (64.2%)  325 (35.8%) | 438  275 (62.8%)  163 (37.2%) | 471  309 (65.6%)  162 (34.4%) | **0.3755** |
| **Depressive symptoms**  CESD Score < 10  CESD Score >= 10 | 908  230 (25.3%)  678 (74.7%) | 437  104 (23.8%)  333 (76.2%) | 471  126 (26.8%)  345 (73.2%) | **0.3066** |
| **Viral status at baseline**  Not Virally Suppressed  Virally Suppressed (<200) | 770  397 (51.6%)  373 (48.4%) | 299  155 (51.8%)  144 (48.2%) | 471  242 (51.4%)  229 (48.6%) | **0.9011** |
| **Physical composite score** Mean (Std. Dev.) Min, Median, Max | 908 37.9 (12.3) 5.5, 38.9, 66.6 | 437 37.9 (12.4) 5.5, 38.9, 66.6 | 471 37.9 (12.2) 6.8, 39.0, 63.8 | **0.9725** |
| **Mental composite score (MCS)-quality of life** Mean (Std. Dev.) Min, Median, Max | 908 34.8 (12.9) 0.6, 35.4, 66.3 | 437 33.8 (12.8) 0.6, 34.0, 64.7 | 471 35.8 (12.8) 2.3, 36.4, 66.3 | **0.0194** |
| **Time living with HIV in years** Mean (Std. Dev.) Min, Median, Max | 896 11.0 (8.8) -0.6, 9.5, 35.9 | 425 10.8 (8.6) 0.0, 9.3, 35.9 | 471 11.2 (9.0) -0.6, 9.6, 35.9 | **0.4960** |
| **Years homeless**  Mean (Std. Dev.) Min, Median, Max | 900 6.5 (8.5) 0.0, 3.0, 55.0 | 429 6.9 (9.0) 0.0, 3.0, 55.0 | 471 6.1 (8.0) 0.0, 3.0, 50.0 | **0.1835** |
