# Supplementary material for: Pathways to housing stability and viral suppression for people living with HIV/AIDS: Findings from the Building a Medical Home for Multiply Diagnosed HIV-positive Homeless Populations initiative
Source: PLoS One. 2020 Oct 1;15(10):e0239190. doi: 10.1371/journal.pone.0239190 (PMC7529314; doi:10.1371/journal.pone.0239190)
Supplement: Attachment — Submitted filename: Response to Reviewers-4-2020.docx [file pone.0239190.s002.docx]

61 Wilder Street O’Leary Library, Suite 540

Lowell, Massachusetts 01854

tel:	978.934. 4515

fax:   	978.934.3012

**ZUCKERBERG College of Health Sciences**

*Department of Public Health*

April 18, 2020

Editorial Board

*PLOS ONE*

Dear Editors,

I am pleased to submit revisions to our manuscript (PONE-D-19-29536] entitled “Pathways to housing stability and viral suppression for people living with HIV/AIDS: Findings from the Building a Medical Home for Multiply Diagnosed HIV positive Homeless Populations Initiative” submitted as part of the HRSA Collection. Thank you for the suggested revisions to our manuscript. Below I provide a response to the editorial comments and each reviewer’s comments received on February 4, 2020.

We have reviewed PLOS One’s style requirements and updated the main body of the manuscript and formatting for the title and authors. The Ethics statement in the submission form has been revised to be the same form in the manuscript and now includes the full names of the ethics committee/institutional review board (s) for the local study sites (lines 127-133) as well as the multisite evaluation center at Boston University.

Finally, with respect to making the data available, we state that *the data are available upon request* due to the sensitive nature of the data and as required by our IRB approval. Contact information for the name of the Principal Investigator (Serena Rajabiun, PhD (rajabiun@ bu.edu) in order to access de-identified datasets.

Response to Reviewer 1

- HRSA’s policy for written communications, including manuscripts requires that we use “people with HIV” and avoid using acronyms such as “PLWH”.
- We have added appropriate titles and legends to Figures 1 and 2. Figure 1 visually depicts our framework for the study. The title was somehow omitted when embedded in the manuscript and now has been added. In Figure 2, there is no legend associated with the path model. However, we revised the Figure to avoid acronyms such as “AOR” and changed to “adjusted odds ratio”.
- The abstract has been revised to ensure consistency with the tables and the manuscript. Table 1 shows the unadjusted bivariate analysis that select the criteria variables for the final path analysis. In unadjusted analyses, gender was a significant factor associated with being stably housed at 6 months and retention in care and therefore, included in the final analytic model. Male gender is the reference group and our findings indicate that transgender persons were *significantly less likely to be stably housed at 6 months* compared to males, while females were more likely to be *retained in care at 12 months* compared to males. The abstract has been revised to exclude the reporting of the unadjusted bivariate analyses and only include the findings from the final path model.
- Depression screening: we have updated the methods section to include that we used the short form 10-item Centers for Epidemiologic Studies-Depression Scale.
- Findings related to patient navigation and viral suppression: Thank you for your comment about the surprising negative finding that persons who received higher doses of patient navigation were less likely to be virally suppressed in the final analysis. We have provided more detail in the methods and discussion section about how patient navigation was conducted based on the findings in Tables 1 and 2 and the final path analysis in Figure 2. We hypothesize that patients with whom patient navigators were providing a greater frequency of contact and variety of support services (health care, behavioral health support, other social service needs) had other priority needs. We separated our initial Table 1 into two tables: Table 1: Sociodemographic and health characteristics of the study sample and Table 2: Type and Intensity of Navigation Activities by Housing and Health Outcomes. Our findings in Table 2 show that navigators provided a significant amount of health care related services which included education and support with medications. One of the limitations of our study is that we did not collect data on whether patient navigators specifically observed patients taking medications which would more directly lead to viral suppression. It was also beyond the scope of this study to tease out if participants who received navigation support with obtaining medications had also received directly observed therapy compared to those who did not have better viral suppression. Our encounter form for navigators only documented “assist with obtaining medications”. This is an area for further study.
- Thank you for the suggestion to review the studies published by HL Cooper and O Galarraga. We conducted a specific literature review to focus on people experiencing homelessness and HIV. We added the relevant studies from Galarraga and other recently published in 2018.

Reviewer 2

- For the abstract we added a definition of patient navigator and a description of the sites to give more context for the study (lines 29-30). In addition, we added a dot marks when appropriate. Thank you for these edits.
- In line 54 we revised the sentence to mention that interventions address the unmet social and health needs for this population and not only biomedical interventions.
- **Introduction:** We revised the section to elaborate that while there is ample evidence that unstable housing affects HIV health outcomes, evidence is needed on the types of interventions and policies and the mechanisms of how they address housing instability and health outcomes. We also revised this section so it clearly addresses the gap in the research: examining how patient navigation can affect housing stability and viral suppression and if there is a direct pathway.
  - Added a description of a navigator model (lines 68-71).
  - Added the objective of the study to clearly state that we are examining the mediating effect of housing stability on health-related outcomes (lines 82-86).
  - The analyses for this study use outcomes based on HRSA core indicators of viral suppression at 12 months and retention in care at 12 months. Therefore, participants were enrolled in the study and followed for a minimum of 12 months and sites were required to collect data on participants at least to 12 months post enrollment.
- **Methods:** We added more description about location and organizational settings for the nine sites. We had cited the original study which provides this background information to save on word count. We also added more description about the patient navigation model and the types of services, specifically housing services that were provided. Our encounter form collected information about housing search, accompaniment to housing support services and rental assistance, emergency housing services for motel stays; move in support. Our program intervention did not directly provide rental assistance but more navigators helped to connect participants to these programs including housing vouchers and other subsidy programs.
  - Lines 108-112: we revised to state that across the project 909 participants were enrolled and this study focuses on the subsample with 471 with complete housing and HIV health outcome data up to 12-month post intervention.
  - Line 114-116: Our criteria used to define “unstably housing status” is based on HUD’s definition of homelessness and unstable housing. We have provided the citation and updated the manuscript to include that description.
  - Unmet need for services includes both social and other non-medical needs as well as medical needs (lines 168-169)
  - We have updated our analysis to only focus on the sample with complete data n=471 (line 108). We have also included a statement about the difference between this sample and those enrolled (n=438) (See lines 325-332) who did not have complete data on housing stability or health outcomes due to being lost to follow up at 6 or 12 months. We found the samples to be similar on sociodemographic variables (age, race/ethnicity, gender, education) but our final analytic sample had fewer social and medical unmet needs and barriers to care, and fewer chronically homeless persons compared to the overall samples with incomplete data (n=438). We have also included a table in the Supplementary Information section describing these differences.
  - Statistical Analyses (lines 176-191): Thank you for this suggestion and citation. Our main objective of the study was to examine housing stability as a mediator on health outcomes. Therefore, we wanted to see if achieving housing stability and dose of patient navigation at 6 months led to better outcomes at 12 months. We used current standard software for path analysis, M-plus, that can incorporate time varying mediation. We realize that it is challenging to achieve housing stability in 6 months. We wanted, however, to examine intervention effects that occurred prior to our outcomes to better understand the potential mechanisms of action of the intervention. If our intervention and outcomes were occurring at the same time point (12 months) it would be more difficult for us to tease out the intervention mechanism.
  - Measures lines 132-174: we have included more information and description about all our measures. We had included a citation to a previous published study that has all the measures defined to save on word count. However, this section is now updated per the reviewer’s suggestion to include all the measures and appropriate citations.
  - Navigation activities lines 141-156: We have included more description of the activities provided under each program domain.
  - Comment #11 on intervention dose: Our goal in this paper was to explore the global effects of patient navigation and dose on housing stability and health outcomes. It was beyond the scope of the paper to examine the types of activities. We wanted to keep the paper focus to the questions of overall dose. We are planning to do a more in-depth exploration for patient navigation activities in this population in the future.
  - Pharmacological viral treatment: We did not collect information on the specific pharmacologic treatments. This paper focuses on navigation and linkage to housing and HIV health outcomes: retention and viral suppression.
- **Discussion:** We have revised the manuscript to give more understanding about patient navigation and who the patients are and the types of services provided. We have also included more description of the unmet needs, both non-medical and medical.
  - Line 307-314: We have revised this section and commented on whether navigation activities facilitate immediate access to housing. In table 3 our logistic regression shows that navigation dose at any level led to greater odds of housing stability, although there was no significant difference between those who received a lower dose versus those who received a higher dose. We more clearly explain that our navigators provided multiple services and it is difficult to tease out if one type of service made a difference on housing stability (see Table 1). In bivariate analyses, types of services seemed to more related to health outcomes viral suppression and health care support and access to housing and retention in care.
- Line 216-233: We have revised to include a description based on bivariate analyses that health care related services and educational and emotional support are more likely to impact viral suppression. There was no one type of service (Table 2) that was more related to housing stability, as it appears all activity was related although we did not find a significant association. This could be attributed to the short term of 6 months time frame that we investigated.
- Line 313-314: Yes, we included the mixed effects of educational support on viral suppression in the manuscript. Please see lines 222-228.
- Limitations: Yes, participants had access to pharmacological treatment. Although we did not collect data on their specific medications—this was a patient center medical home and the role of the navigator was to connect participants to HIV medical care (a prescribing provider) and housing

Reviewer 3:

- Comments #1 & 2: We have revised the abstract and edited for appropriate punctuation such as removing or adding extra periods per the Reviewers 2 & 3 suggestions.
- Comment #3: We have updated the introduction to provide more detailed description of Navigation models.
- Comment #3: Line 77-79, the test for statistical significance has been added
- Comment # 4: Lines 97-101: A general definition of the medical home intervention is included.
- Comment #5: Lines 134-137: The definition for retention in care and viral suppression are defined under measures in the methods section of the paper. The time for both measures is at 12 months post intervention and we have added a citation for both measures. The viral suppression measure is the standard measure used by HRSA. Retention in care has several definitions we have selected to use the one recommended in the paper by Mugavero. From our perspective this definition would seem important for continuity of care for people with HIV who are experiencing homelessness and a co-morbid condition such as mental health and/or substance use.
- Comments # 6 & #7 on Table 1: We have revised the table to include row percentages per the reviewer’s suggestion. In addition, we have clearly labelled all Column headers to include N (%).
- Comment # 8: Thank you for your question about our finding that there was a higher percentage of participants virally suppressed (75%) vs retained in care (for males 75% were virally suppressed and 55% were retained in care). We hypothesize this is attributable to the fact that some of our sites had medical home teams that were “mobile” with a physician, navigator, nurse and/or social worker. Some participants were seen by a prescribing health care provider in the community, were followed up with their antiretroviral medication consistently, and then connected with a four walls clinic such as community health center (with which many of these mobile teams were affiliated or had partnerships). Furthermore, we used a conservative definition of retention in care in which a person had to be seen at the clinic at least two appointment 90 days apart in a 12-month period. Some participants may have only been seen at 6 months and then could have received their ART prescription for 6 months. Unfortunately, we were not able to obtain from chart review the date of last prescription. We only had information if they had a recent prescription documented in their chart (yes/no).
- Comment # 9: We have updated the Gender variable in Table 1 to include the titles: “cis male” and “b cisgender female”.
- Comment #10: lines # 321-326: The manuscript has been revised to more neutral language and we removed the perceived bias towards patient navigation activities. In addition, we have expanded discussion in the second paragraph about the lack of randomization in the study. Unfortunately, we were not able to find a citation about the higher need patients needing the most help and struggling to achieve housing stability and viral suppression.
- Comment #11: The sentence has been revised to say “across” instead of “crossed”. Thank you for finding this error.
- Comments #12-14: This section has been revised to remove any reference to peer navigation models so as not to speculate differences with this model versus other navigation models since it was not the scope of the paper. The scope of this paper was to examine dose of navigation activities and a future paper will examine the team composition.
- Comment #15: The sentence has been changed from “for” to “of”.
- Comment #16. The results section (lines 257-255) indicate the findings for transgender and food insecure persons.

Thank you for the detailed review of our manuscript and the opportunity to make revisions. We look forward to the opportunity to publish our study in PLoS ONE.

Sincerely,

Serena Rajabiun, PhD
